# ACO-optimized MobileNetV2-ShuffleNet hybrid model for automated dental caries classification

**DOI:** 10.1038/s41598-025-24375-z

**Published:** 2025-11-18

**Authors:** Kotturu Kaveri, Venkata Ratna Prabha K., G. Pradeep Reddy, Sree Lakshmi Ganesh Pothamsetti, Kodali Radha, Ramesh Penumaka

**Affiliations:** 1 Department of Electronics and Communication Engineering, Siddhartha Academy of Higher Education, Deemed to be University, Kanuru, Vijayawada, Andhra Pradesh 520007 India; 2 Department of Information and Communication Technology, Manipal Institute of Technology, Manipal Academy of Higher Education, Manipal, Karnataka 576104 India; 3 Division of Pediatric Neurology, Department of Pediatrics, University of Tennessee Health Science Center, Memphis, TN 38103 USA; 4 Department of Conservative Dentistry and Endodontics, Drs. S&NR Siddhartha Institute of Dental Sciences, Chinaoutpalli, Andhra Pradesh 521286 India

**Keywords:** ACO, Dental caries, MobileNetV2, Panoramic radiographs, Sobel-Feldman edge detection, ShuffleNet, Computational biology and bioinformatics, Diseases, Engineering, Health care, Mathematics and computing, Medical research

## Abstract

Dental infections may result in severe health conditions when not diagnosed and responded to immediately. However, it is a difficult process that can take time and expertise to diagnose oral infections based on X-ray images. In this paper, a new method of dental caries classification based on the panoramic radiographic images is proposed, which is aimed at overcoming the class imbalance and weak anatomical differences. During the preprocessing stage, the clustering technique was used to form similar grouped data to balance the distribution of data, and the Sobel-Feldman edge technique was applied to emphasize critical features. MobileNetV2 and ShuffleNet models were also trained on the preprocessed set of data separately, but the classification ability was poor. A hybrid architecture was designed based on the combination of the strengths of the two models, so the level of precision increased. In a further effort to improve the performance of the model, Ant Colony Optimization (ACO) algorithm was incorporated to the hybrid framework. Addition of ACO made the classification highly accurate since it could perform an efficient global search and parameter tuning. The suggested ACO-enhanced hybrid approach showed better results with 92.67% accuracy than standalone networks which implies that the proposed model can be used on reliable and automated dental diagnosis.

## Introduction

Bacteria interacting with sugar creates tooth damage that leads to cavity formation. Tooth cavities spread widely throughout the population because more than 90% of individuals age 20 or older develop at least one cavity during their lifetimes^1^. During early detection, dentists use physical assessment techniques to examine patients. Medical personnel determine the extent of tissue damage through manual testing via periodontal probes. Each tooth receives manual probing which reveals periodontal disease severity while determining its progression by focusing on the most affected area. People experience pain along with discomfort during this complex diagnostic procedure^2^. Dental cavities become visible as distinct features when radiographic images are analyzed. Therefore, most dental professionals use X-ray images, such as intraoral and panoramic radiographs, as their primary diagnostic tool. Recent advancement in the field of artificial intelligence (AI) now serves as an efficient system for both dental caries classification and segmentation^3^. Deep learning (DL) applications now utilize new pattern recognition capabilities within medical imaging data. Recent studies focus on pattern recognition since dental cavity detection represents a fundamental pattern recognition task. Artificial neural networks (ANN), together with convolutional neural networks (CNN), enable fast and accurate dental problem evaluation^4^. Hybrid neural networks combine ANN and Deep neural networks (DNN) structures to extract hidden data attributes through stacked sparse autoencoders and refine these features using logistic regression classifiers for output enhancement. Using conventional DL methods can be very difficult since they require both strong computing power and a lot of memory, limiting how they are used on small devices like smartphones, embedded systems, and IoT gadgets. Due to this issue, people are searching for efficient neural network structures that give good accuracy and low computational cost. Therefore, models are required to meet both large processing needs and requirements for small sizes. One way to address this problem is with EfficientNets, which uses a compound scaling approach to create its models. Traditional models change depth, width, or resolution, while EfficientNet uses a series of fixed scaling coefficients to change these features properly. This method supports smoother running of applications in different computing environments because it improves how resources are managed^5^. Yet, traditional neural networks encounter various problems in optimization and adaptation, mainly in situations that involve specific domains. In order to solve these problems, the current study introduces a hybrid model that joins several efficient neural network designs with a nature-inspired optimization method. The suggested technique unites MobileNetV2 and ShuffleNet, recognized for belonging to the top lightweight CNNs, which are easy to use and economical. Also, the ACO algorithm is then applied to these models, since it is based on how ants forage and does particularly well in solving both combinatorial and parameter optimization tasks^6^. Merging the effectiveness of top classifiers with the ability of ACO to search over many instances, the new model seeks to improve the accuracy of classification while still being efficient. Since this hybrid framework works well on low-powered devices, it can be used in real cases like medical imaging, object detection and smart surveillance systems.

This work introduces two significant contributions:

- Enhancing focus on crucial image features by utilizing MobileNetv2 and ShuffleNet for dental caries classification.
- Demonstrating superior classification performance with a hybrid network using Ant colony optimizer.

The structure of the article is as follows: A detailed examination of research on caries classification and detection is given in “Literature”. “Methodology” section outlines the research design and techniques used in this research. The results of the evaluation using the suggested technique are shown in “Result analysis”, which also shows that models reached a noteworthy level of accuracy. The study’s major findings are finally summarized in “Conclusion”, along with their implications for further research in this area.

## Literature

Machine learning (ML) is being used early on for the classification and identification of dental diseases. Especially in caries detection, Random Forest, Decision tree and Logistic Regression are commonly used ML algorithms^7^. For tasks related to image recognition, the supervised learning processes are applied. Adding large datasets of dental issues can better train ML^8^. Over the years, there is shift from ML to DL in medicine and healthcare since traditional ML algorithms (SVM and decision trees) are manual and may fail to detect important patterns due to the need for expertise. DL networks such as Generative adversarial networks (GANs), Recurrent neural networks (RNNs), Deep neural networks are being used for the recent studies^9^.

**Table 1:** Summary of recent approaches for dental infection classification.

Ref	Year	Objective	Dental infection type	Image type	Models used
^10^	2025	A reliable system for classification and detection of caries.	Caries	Bitewing radiographs	U-Net with three encoders: ResNet50, ResNeXt101, and VGG19
^11^	2025	Tooth and patient-level classification and detection of periodontal disease using hybrid classification networks.	Periodontitis	Panoramic radiographs	Classification-H-Net, HC-Net; Visualization-CAM
^12^	2022	Aims to develop a hybrid optimization based approach integrated with deep learning methods for accurate classification of dental caries.	Caries	Radiographic images	Filter-Wiener filter; Segmentation-BWO; feature extraction–-GLCM; classification-CNN
^13^	2021	To detect the caries-affected layer using hybrid neural network	Caries	Bitewing radiographs	Preprocessing-selective median filter; classification-HNN (ANN + DNN)

A U-Net model and three parallel ResNet50, ResNeXt101 and VGG19 encoders are used to allow the model to find patterns and details at many possible scales. This integration of various encoder networks means the system correctly and effectively identifies carious lesions, helping doctors to discover and address these problems at a very early stage^10^. A hybrid classification network is utilized for the detection and division of periodontal disease both at individual tooth-level and whole-patient-level. The new framework makes use of two models–H-Net and HC-Net–to label and sort healthy and caries effected teeth hierarchically. The Regions of Interest for periodontal issues are made clearer by using Class Activation Mapping (CAM) when visualizing X-rays^11^. DL models basically works by learning features and this learning success is affected by hyper parameters which include the learning rate, batch size and the number of layers^14^. In recent years, applying CNN-based algorithms in DL to image analysis has achieved effective results, making these algorithms widely used in finding caries^15^. After a major breakthrough in this technology, hybrid deep learning approach is introduced to enhance the image recognition capabilities by combining efficient neural networks to the basic DL networks^16^. A hybrid model that uses test-time augmentation and describes deep transfer learning was developed to identify prone-to-cavitation teeth from non-standardized photographs, with acceptable clinical results^17^. To enhance performance, optimizers are also included to hybrid models. DL models, as opposed to traditional gradient-based methods, employ optimizers to increase the rate of convergence, avoid local minima, and more effectively modify hyper parameters. They use strategies seen in nature to cover the whole search area and advance the model’s functionality. The three critical steps in the effective development of dental caries diagnosis and treatment models include classification, identification (detection), and segmentation. Classification is used to assess the nature and extent of caries, detection is used to confirm their existence in radiographic images, segmentation is used to outline areas of interest in a targeted clinical intervention^18^. This work is concerned with the classification and identification of dental caries presence or absence. Table 1 represents the summary of recent works done in the field of dentistry for then dental infection classification.

**Table 2:** Summary of recent approaches for dental infection detection.

Ref	Year	Objective	Dental infection type	Image type	Models used
^19^	2024	To improve deep learning-based recognition of dental diseases in X-ray images collected from hospitals.	Six major classes: healthy teeth, caries, impacted teeth, infections, fractured teeth, and broken-down crowns/roots (BDC/BDR).	Panoramic dental radiographs	Image enhancement-contrast-limited adaptive histogram equalization (CLAHE); identification-YOLOv7
^20^	2024	To propose effective methodology for segmentation and classification of tooth types in 3D dental models	–	Panoramic dental X-rays	Preprocessing-random number image conversion filter; segmentation-custom mask-CNN; classification-adaptive enhanced GoogLeNet (AEG)
^21^	2022	To utilize the deep gradient-based LeNet classifier model for the diagnosis of caries lesions.	Caries	Bitewing radiographs	Preprocessing-Gaussian filter, gray scaling, thresholding; feature extraction-kernel-based non-linear fisher analysis; classification-DGLeNet
^22^	2022	To enhance caries detection using FOC-KKC segmentation and metaheuristic-based ResNeXt-RNN.	Caries	Periodic dental X-ray images	Preprocessing-CLAHE, bilateral filtering; segmentation-FOC-KKC with hybrid sea lion–squirrel search optimization (HSLnSSO); detection-M-ResNeXt-RNN with HSLnSSO

Table 2 represents the summary of recent works done in the field of dentistry for the dental infection detection. YOLOv7 is applied to diagnose and classify these conditions, providing outstanding and precise recognition for many dental threats. It is intended to accurately distinguish and assign six main dental problems: healthy teeth, dental caries, impacted teeth, infections, fractured teeth and broken-down crowns or roots (BDC/BDR)^19^. A unique way to segment and classify tooth types from 3D models made from panoramic dental X-rays. Before further analysis, a random number-based filter on images is used to improve the image quality and prepare the data. The segmentation of teeth in X-rays is carried out using a custom Mask-CNN. An AEG model is employed which can accurately recognize different tooth types in both simple and complicated dental structures^20^. A new method that uses both optimization and deep learning is introduced to divide the caries-involved areas with a Bat Whale Optimization (BWO) model. Preprocessing is done with a Wiener filter in the suggested framework to decrease image noise and improve quality.

The Gray Level Co-occurrence Matrix (GLCM) is then used to identify key texture patterns. Lastly, a CNN is used for precise classification and the combined BWO-CNN structure results in improved overall diagnostics^12^. A LeNet (DGLeNet) classifier is used to identify caries lesions in digital bitewing radiographs. In preprocessing step, techniques such as Gaussian filtering, grayscale transformation and thresholding, are used to better shape and distinguish the main features in an image. Kernel-based nonlinear Fisher Analysis is applied to find features that help distinguish one class from another. DGLeNet is then applied to identify each refined feature category^21^. The approach introduces hybrid dental caries segmentation to increase accuracy and precision in detecting caries in periodic dental X-ray images. The enhancement of the image begins with a CLAHE and ends with applying a bilateral filter. To segment images, a new method using FOC-KKC and HSLnSSO is utilized to find the best results. A HSLnSSO-enhanced version of ResNeXt-RNN is used for detection^22^. To improve accuracy in finding and pointing carious areas, a hybrid neural network architecture integrating both ANNs and DNNs is implemented. At the preprocessing step, a selective median filter is used to lower the noise amount while not harming significant elements of the image^13^. Based on the recent developments, this work proposes feature extraction using a hybrid model and optimizer-guided classification to improve caries classification accuracy.

## Methodology

**Fig. 1:**
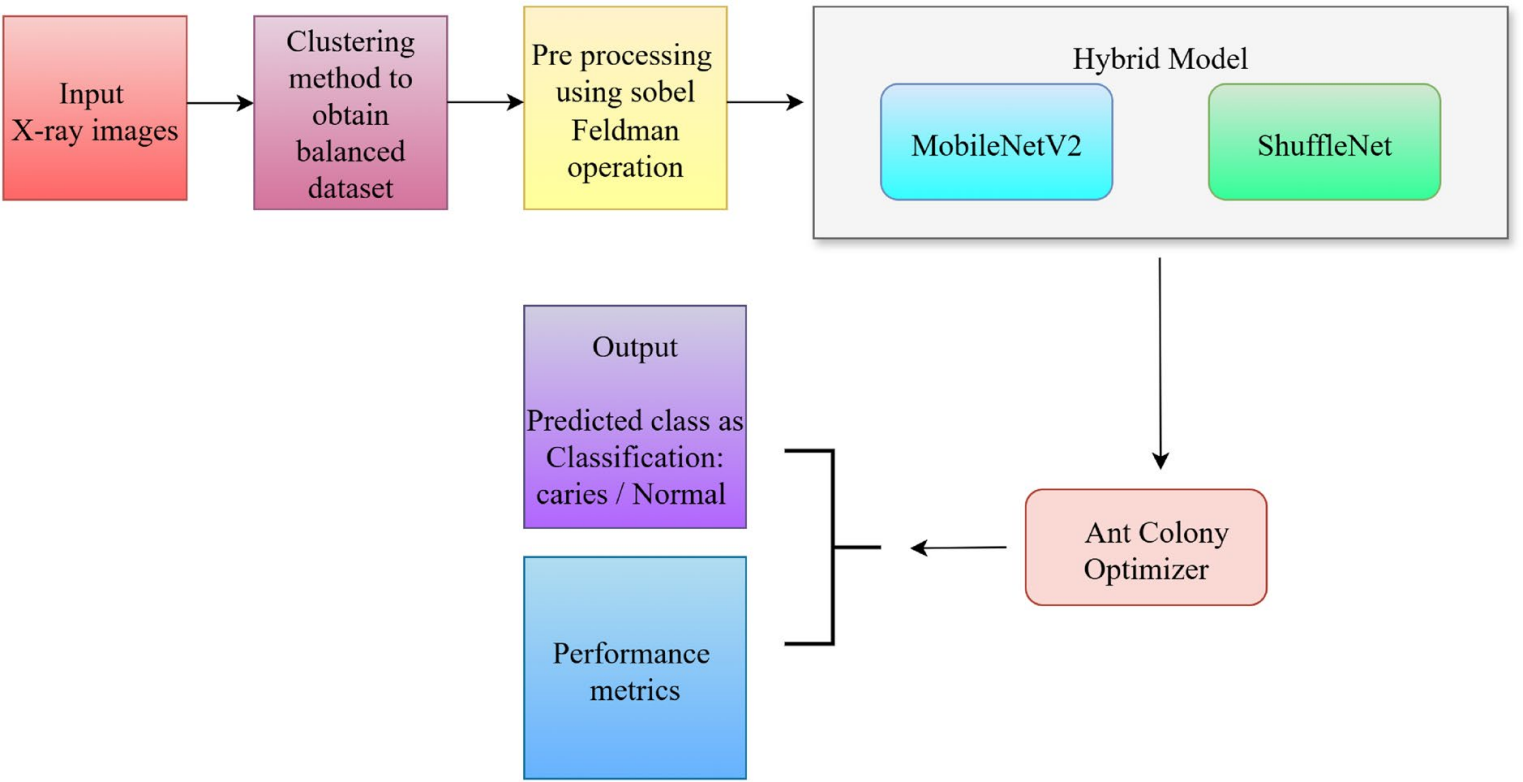
Proposed work flow for dental caries classification.

This work represents a unified pathway of automating the process of classification of caries via X-ray images based on the traditional approaches of image processing and more modern methods of DL and optimization, as shown in the Fig. 1.

First, the clustering-based approach is used to balance input dental radiographs to overcome the problem of class imbalance and then preprocessing is performed using Sobel-Feldman operator so as to improve edge features. The images so enhanced are then fed to a hybrid model of deep learning (MobileNetV2 and ShuffleNet) working in parallel so as to have extractors of rich and diverse feature representations. The next stage involves optimizing/filtering the most discriminative features and this is done through the ACO using its bio-inspired search procedure. The features that underwent the optimization process are then labeled caries or normal and the performance of the same model is assessed according to standard measures to determine its robustness and the ability to produce a reliable diagnosis.

### Dataset information

The initial dataset consists of 13000 images, which includes 3069 images with caries. A clustering-based selection^23^ method was applied to select 3069 non-caries images out of the total remaining 9,931 images to ensure a balanced dataset. K-means algorithm clustering is a way of dividing the minority class into groups based on the distribution regulation within it and the similar samples, forming groups for each of them, and creating new in these samples^24^. First, all the non-caries images are fed through a pre-trained ResNet-18 to get 512-dimensional feature vectors at the ’pool5’ layer. Then, each feature vector goes through K-means which creates up to 100 clusters for similar images. From each group, an amount of images is chosen randomly so that the whole selection has a wide range of differences. This prevents random sampling bias and makes sure that variance is found within the same category. Images with caries are kept in full, whereas only a limited number of non-caries images remain after the selection. Consequently, there are equal or almost equal numbers of caries and non-caries images which is perfect for using deep learning to solve binary classification problems.

### Data preprocessing

Before analyzing an image, preprocessing turns it into a form that makes it easier for the model to understand^25^. Common operations in image processing are resizing, converting to grayscale, reducing background noise, adjusting maximum pixel values to be the same throughout the images and finding the edges to help show important features and make sure all images are related^26^.

**Fig. 2:**
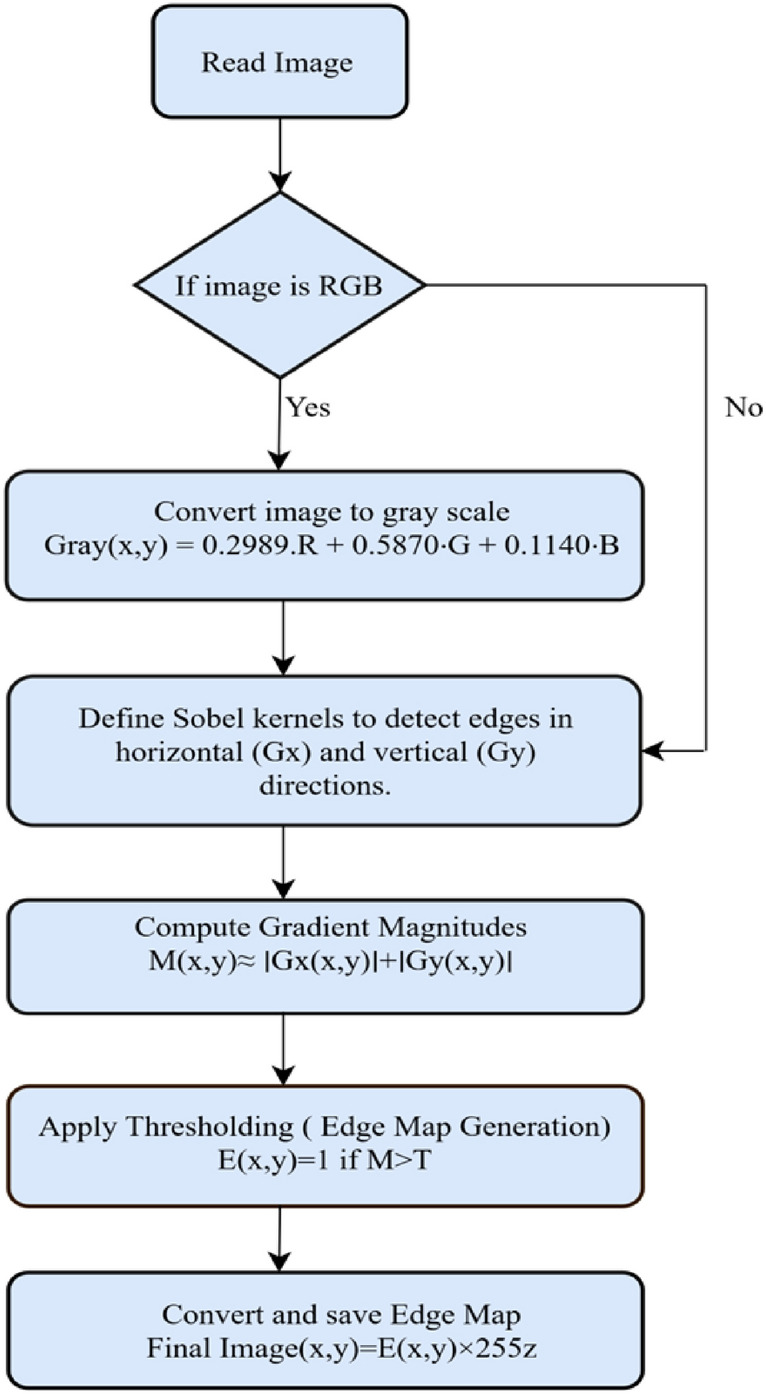
Sobel edge detection algorithm.

In this work Sobel-Feldman operation is used. Digital images have intensity functions defined only at specific points, so the direct calculation of derivatives depends on assuming a smooth continuous and differentiable intensity function hidden behind the sample points. Under these assumptions and extra rules, the derivative of the continuous function can be estimated using the discrete data from the image. In most cases, these methods use the average of intensity in a wide region, but in practice, calculations over small local areas are more common. Some examples of such methods include the Sobel-Feldman operator which gives a coarse estimate of the image gradient^27^. It determines the gradient only by considering the intensities of the pixel and eight surrounding pixels and using simple numbers to assign importance to each intensity. Its low precision does not matter much because it is computationally light and still sufficient for a wide range of real-world image processing applications^28^. Sobel edge detection highlights the edges of teeth, caries regions, and structures in X-rays, helping to aid in segmentation and classification. Fig. 2 represents the algorithmic steps applied on the image using Sobel edge operator.

### MobileNetV2

The patterns found in X-ray dental images tend to be complex (e.g. structures of teeth, bone density, decay, periodontal diseases). These features are effective to retrieve through the depthwise separable convolutions in MobileNetV2. Its depthwise component deeply filters channels in an element wise manner and may recognize gross patterns such as edges and textures in the X-ray. Such filtered features are then multiplied together point by point to construct more intricate representations that are meaningful to dental pathologies using the subsequent pointwise convolution. Such efficacy is important in handling possibly thousands of X-ray images within a short time.

In contrast to the traditional residual block that links the layers having many channels, the inverted residual block linked by MobileNetV2 connects the thin bottleneck layers^29^. These low dimensional bottleneck layers contain shortcut connections between them. The design itself is memory-efficient and enhanced the gradient propagation between several layers. The network accepts low dimensional, compressed representation^30^. First, this input is extended in dimension. The features that are produced are then filtered with a lightweight depthwise convolution. Most importantly, these characteristics are then mapped back to a low-dimensional space by a linear convolution (i.e., no non-linear activation function). These narrow bottleneck layers are important in having non-linearities (such as ReLU) removed. It has been demonstrated experimentally that non-linearity in bottlenecks can cause a poor performance and it is reasoned that non-linearity is capable of destroying information in the low dimensional spaces. The design process is useful in retaining the representational power. MobileNetV2 makes very heavy use of depthwise separable convolutions, originally provided in MobileNetV1. Such factorization decomposes an ordinary convolution into two layers:

- Depthwise convolution: a single convolutional filter can be applied to each input channel, and does lightweight filtering.
- Pointwise convolution (1x1 convolution): New features are constructed as the linear combinations of the input channels.

This factorization greatly makes an inference on reducing the computation cost of a regular convolution operation. The intermediate layer in the inverted residual block increases the input to a higher level. This expansion layer uses depthwise convolutions that are lightweight and helps to filter features and offers non-linearity^31^.

### ShuffleNet

ShuffleNet architecture draws its inspiration from group convolution, skip connections, and depthwise separable convolutions. ShuffleNet suggests a group of convolutions, pointwise convolutions with residual path-based shortcuts. It proposes two important operations to perform large computation reduction with high accuracy: pointwise group convolution and channel-shuffle^32^.

**Fig. 3:**
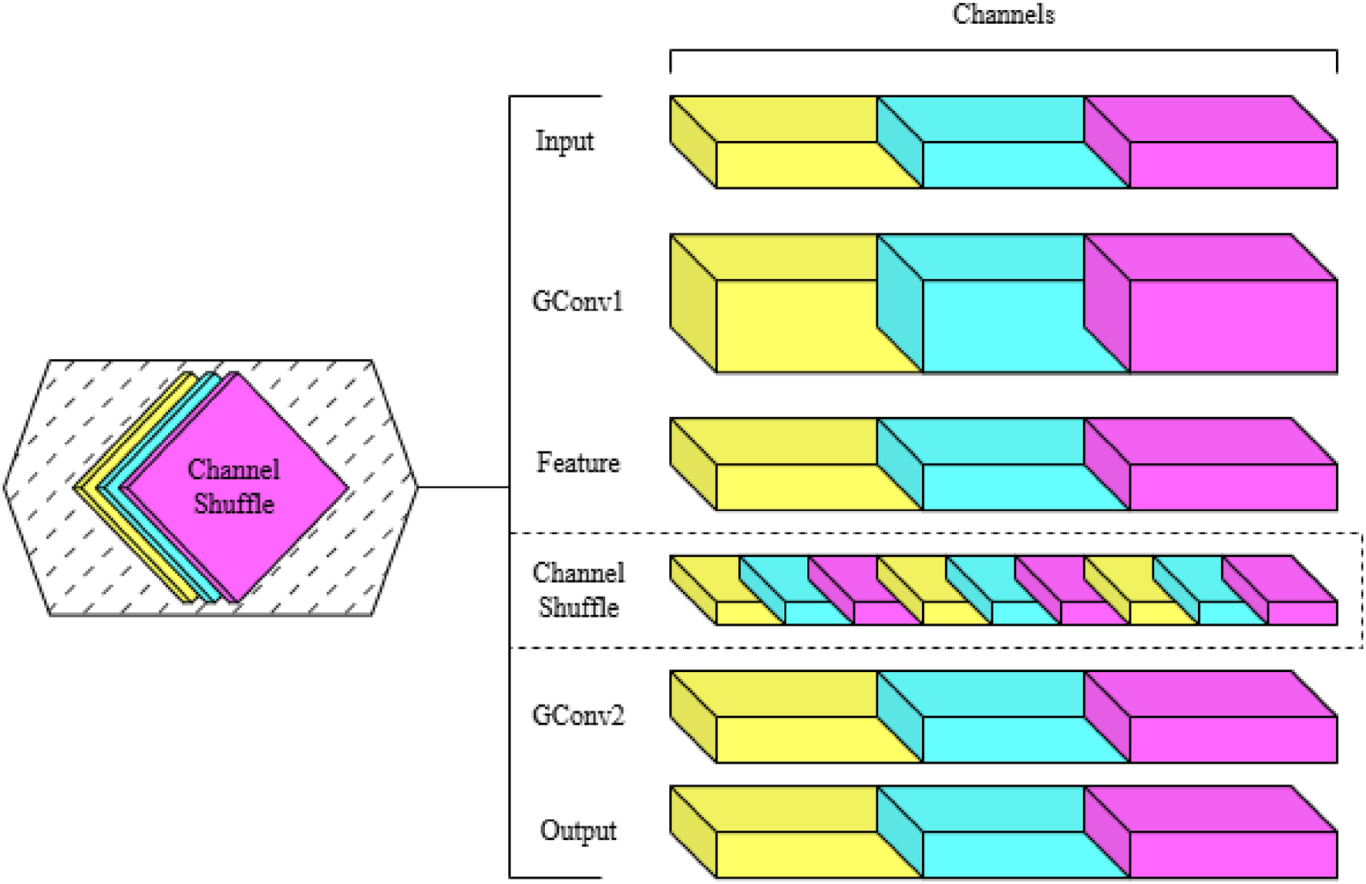
Representation of how channel shuffling takes place in the shuffleNet architecture(Gconv- Group convolution)^32^.

Group convolution involves subdividing the channels of the input into groups, and doing convolutions between each of the groups independently. This enables ShuffleNet to consume a broader amount of feature map channels within the same computational cost, which is essential to enable small networks to encode more representation information. One disadvantage of successive group convolutions is that it can be used to block information flow between channel groups. All the input channels would be used in providing a small subset of output channels belonging to a particular group. As a revocation to this, ShuffleNet introduces the channel shuffle operation. The channels between the groups are re-arranged such that there is a possibility of flow of information through all the channel groups^33^. The Fig. 3 represents how the channel shuffling takes place.

### Feature extraction using hybrid neural network

**Fig. 4:**
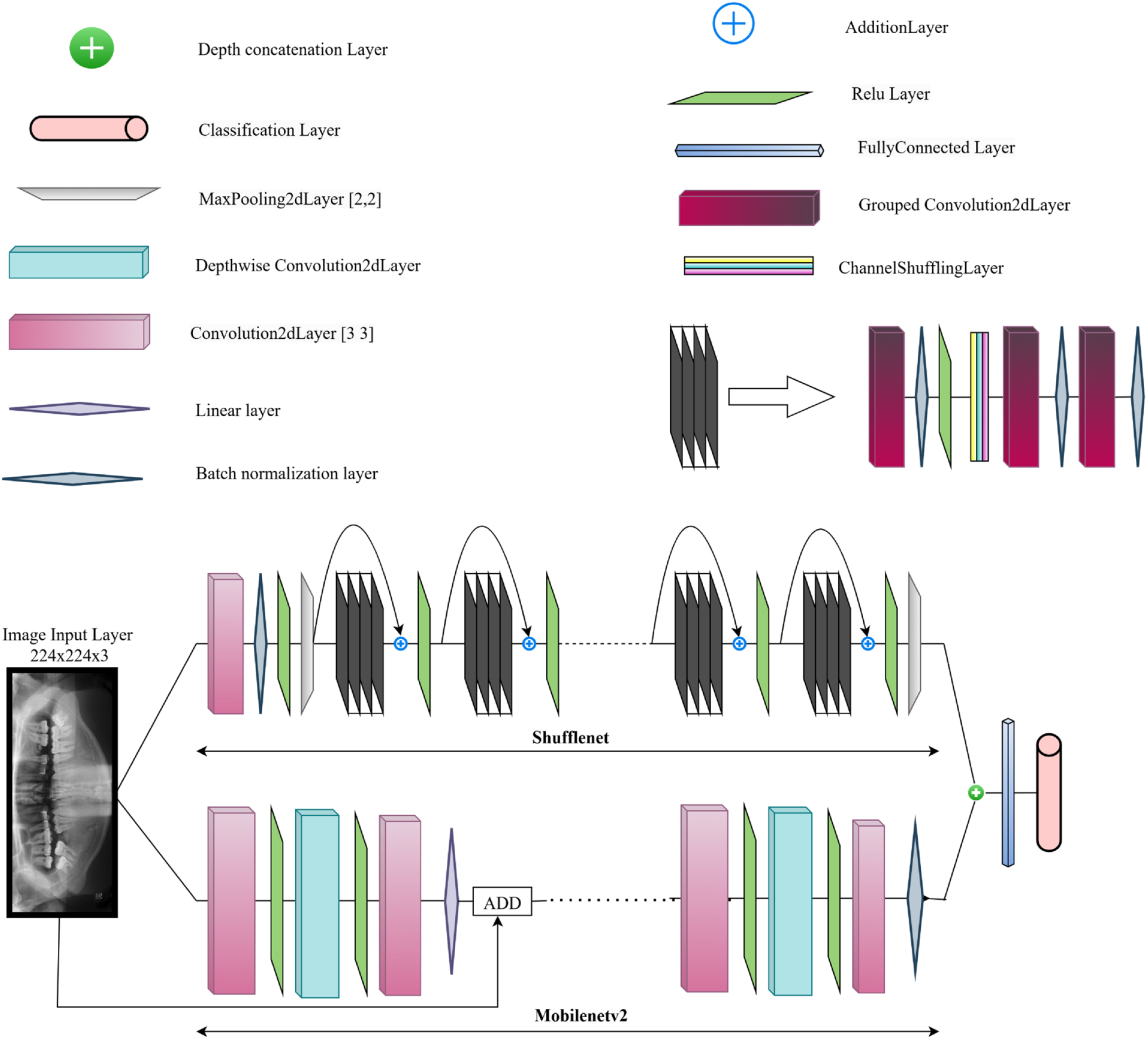
Architecture of the proposed hybrid model.

This work introduces a hybrid network using MobileNetV2 and ShuffleNet as shown in Fig. 4. Starting with this point, the X-ray image is transmitted to two different feature extraction backbones at the same time: ShuffleNet and MobileNetV2.

The ShuffleNet stream (upper path) employs grouped convolutions, channel shuffling, batch normalization and ReLU activation in order to keep the computational complexity low and maintain the representational power. Main factors like depth concatenation layers and addition layers have been implemented to make the feature diffusion and gradient flow better. To guarantee the cross-group information exchange, necessary in the grouped convolutions, the channel shuffling layer is introduced.

MobileNetV2 pathway (lower branch) uses depthwise separable convolutions and linear bottlenecks. MobileNetV2 employs inverted residual, that is, run through pointwise convolutional layer to expand a narrow input to a higher-dimensional feature representation, through depthwise convolutional layer connecting to a compression pointwise projection. This block architecture is effective in edge devices and it facilitates computation. Addition layers and the batch normalization helps stable learning and improved feature refinement. The ShuffleNet and MobileNetV2 branches outputs are concatenated through depth concatenation layer to combine different features of the two branches of the model. The combination of lightweight and high efficiency characteristics of MobileNetV2 and those of small and perfectly mixed feature maps of ShuffleNet enhance the representational capability of this convergence. The fused feature map is then fed into fully connected layers along with linear layer and classification layer, after which the result gives the final predictions.

### Ant colony optimizer for hyperparameter tuning

Inspired by the behavior of an ant colony to seek the shortest path of food, ACO algorithm has become one of the most well-liked swarm-based algorithms^34^. By raising the size of the problem and shape of the search space, the complexity of the search space is raised. Multi-objective functions are the most realistic in terms of optimization problems required to be attained. It is referred to as a multi-objective optimization problems (MOPs)^35^ and this kind of problem has been encountered in various fields, e.g. bioinformatics, medical image analysis, and so on. These optimizers help to fine-tune the hyperparameters to give the best outcomes. In this work, the diagnostic process for caries conditions is influenced by the optimization of hyperparameters of MobileNetV2 and ShuffleNet. ACO deploys artificial ants to traverse along a solution space with the guidance of pheromone traces and heuristic data. The Algorithm 1 works in the framework of a probabilistic decision-making process in which the probability $P_{ij}$ of hopping between node $i$ and node $j$ is subject to the quantity of pheromone $\tau _{ij}$ and heuristic desirability $\eta _{ij}$^36^.

1 $$\begin{aligned} P_{ij} = \frac{[\tau _{ij}]^{\alpha }[\eta _{ij}]^{\beta }}{\sum _{k \in N_i} [\tau _{ik}]^{\alpha } [\eta _{ik}]^{\beta }} \end{aligned}$$

In this case, the relative weight of pheromone against heuristic values is controlled by $\alpha$ and $\beta$, and $N_{i}$ represents the neighborhood of node $i$. Ants build solutions through iterations and jointly consult the pheromone trails to support good routes. Evaporation of pheromone is utilized so that converging too soon is discouraged, and exploration is promoted.

The pheromone update rule is generally expressed as^36^:

2 $$\begin{aligned} \tau _{ij}(t+1) = (1 - \rho ) \cdot \tau _{ij}(t) + \Delta \tau _{ij}(t) \end{aligned}$$

**Algorithm 1:**
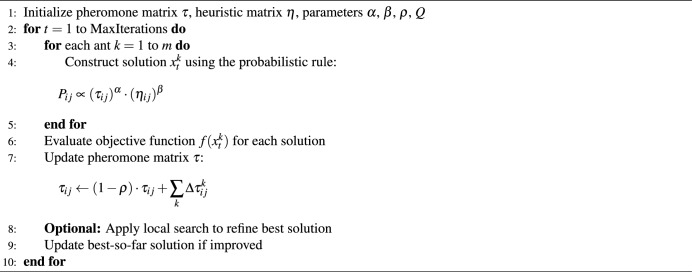
Ant Colony Optimization (ACO)

where $\rho \in (0,1)$ is the evaporation rate, $m$ is the number of ants, and $\Delta \tau _{ij}^k$ is the pheromone deposited by the $k{\text {th}}$ ant. The algorithm aims to converge to the optimal solution $x^*$ such that^36^:

3 $$\begin{aligned} x^* = \arg \max _{x \in S} f(x) \end{aligned}$$

ACO was applied to the hybrid model, to optimize the hyper parameters, and increase the model convergence. Ten ants and 10 iterations are used in the implementation of ACO to balance computational cost and optimization performance. ACO saves on computation cost by probabilistically directing the search using pheromone trails and heuristic data. In contrast to exhaustive grid search which considers all the possibilities, ACO reduces the search space effectively. ACO is more computationally efficient than random search because compared to random search, better solutions paths are reinforced more quickly using fewer evaluations^37^. In particular, it iteratively optimized combinations of learning rate, momentum, and batch size, using simulated ants, following pheromone to optimize the combination of learning rate, momentum, and batch size. The optimization goal was that of maximizing the validation accuracy and minimizing over-fitting.

## Result analysis

This section analyzes the performance of hybrid model with and without optimizer in classifying dental caries in Panoramic X-ray images. It includes comparison between the pre trained models and hybrid model in terms of evaluation metrics. The data set is divided into 7:2:1, and here one part of the whole dataset is used for testing.

### Performance analysis of MobileNetV2

**Fig. 5:**
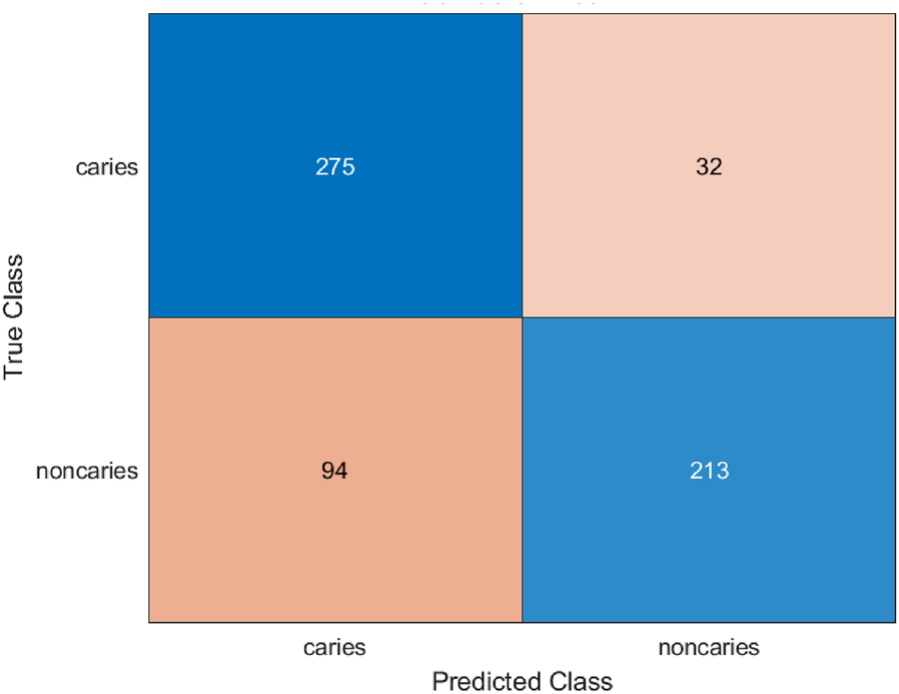
Confusion matrix for MobileNetV2 model.

**Fig. 6:**
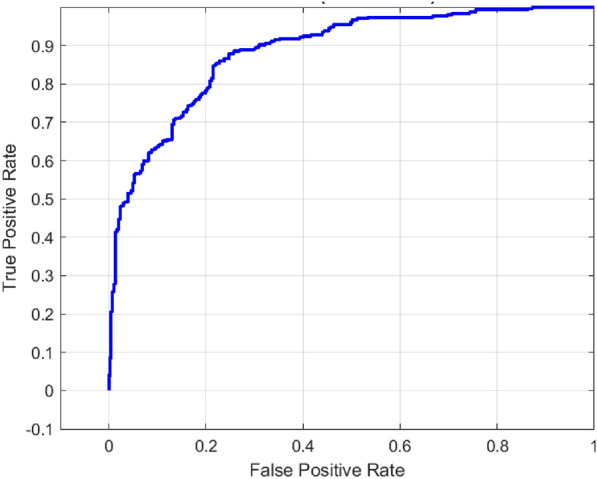
ROC curve for MobileNetV2 model.

Evaluation metrics like accuracy, precision, recall, F1 score, sensitivity, Area Under the Curve (AUC) are calculated. The Fig. 5 , Fig. 6 represents the confusion matrix and ROC curve of the pretrained MobileNetV2 architecture.

The confusion matrix showed that 275 caries and 213 non-caries were classified correctly and 32 caries and 94 non-caries were incorrectly classified. These findings reveal that the model exhibits high sensitivity, but its false positive classification rate is comparatively large when it comes to the non-caries category. This has been supported by the Receiver Operating Characteristic (ROC) curve which has an AUC of 0.886 indicating the high rate of discriminative capability. This steep takeoff at the beginning of the corner of the curve indicates that the model has a robust sensitivity at low false positive rates.

### Performance analysis of ShuffleNet

**Fig. 7:**
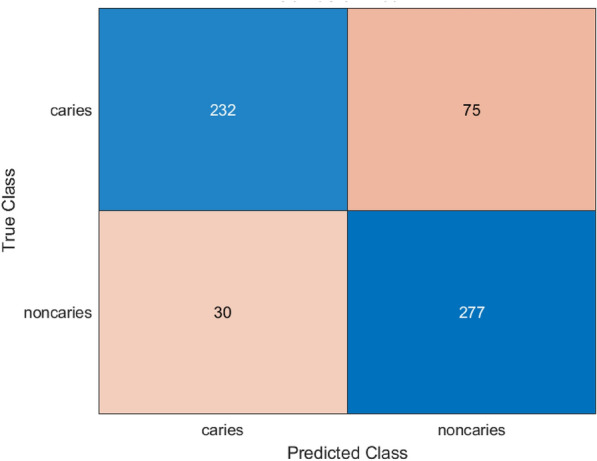
Confusion matrix for ShuffleNet model.

**Fig. 8:**
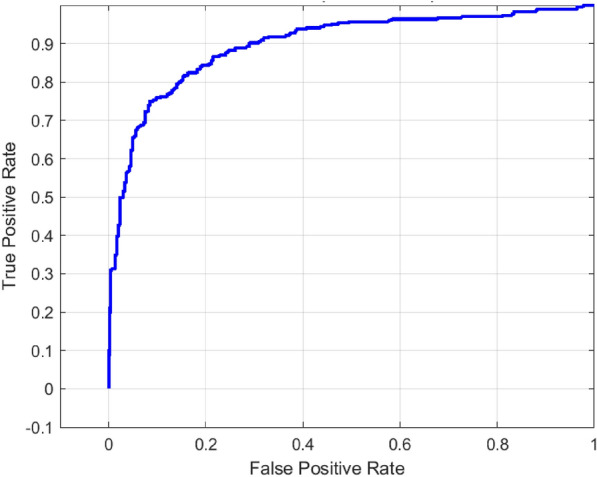
ROC curve for ShuffleNet model.

The model based on ShuffleNet showed good performance in classification, which is shown by the analysis of the confusion matrix in Fig. 7 and the ROC curve in Fig. 8. It absolutely classified 232 caries and 277 non-caries, was false positive in 30 cases (non-caries falsely designated as caries), and false negative in 75 cases (caries falsely designated as non-caries). This indicates that the model has a strong probability of identifying non-caries samples but has an average tendency of identifying all caries samples.

The Table 3 shown represents comparison between the evaluation metrics of the two pretrained models.

**Table 3:** Comparison between final performance metrics of the MobileNetV2 model and ShuffleNet.

Metrics	MobileNetV2 values	ShuffleNet values
Accuracy	0.7660	0.82899
Precision	0.86939	0.78693
Recall	0.69381	0.90228
F1-Score	0.77174	0.84067
AUC	0.88605	0.89916
Sensitivity	0.69381	0.90228

### Performance analysis of proposed hybrid model

**Fig. 9:**
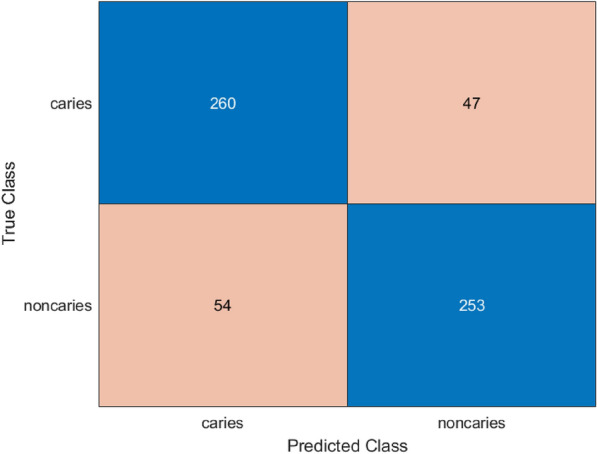
Confusion matrix for proposed hybrid model.

**Fig. 10:**
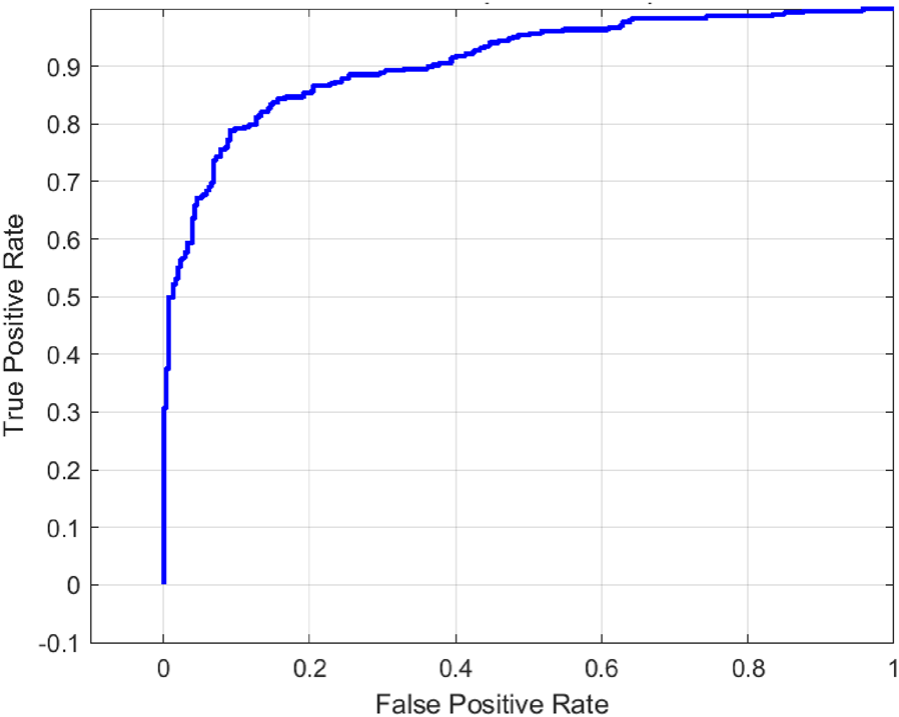
ROC curve for proposed hybrid model.

In order to improve the ability of the features retrieved by MobileNetV2, and ShuffleNet, a fusion network was used. The classification results achieved with the use of the improved feature set are depicted with the help of the confusion matrix shown in Fig. 9. The model demonstrates better balancing between the expected and observed labels of caries and healthy individuals, along with a significant decrease in the number of misclassifications in contrast to the initial model. The model is able to discriminate between the two classes, as also revealed in the ROC curve provided in Fig. 10. The hybrid model had an AUC of 0.909 which has high separability and high consistency of classification.

The hybrid model demonstrated moderate result in the classification of dental X-ray images with a total accuracy of 83.55 percent as shown in Table 4. Based on the confusion matrix the model was able to correctly predict 260 caries and 253 non-caries samples but failure to predict 54 non-caries and 47 caries cases. The F1-score was observed to be 83.36%, this is achieved through recording precision of 84.33% and recall of 82.41%, which indicates an optimum trade-off between false and negatives as well as false positives. Besides, the sensitivity of the model was very high at 98.24%, which means that the skill at identifying the true positive cases (caries) is also high. The ROC curve, as an AUC of 0.90938 proves the high discriminative capabilities of the model, showing robustness of the results in line with different threshold conditions and establishes the hybrid model as a light but powerful architecture in detecting dental disease. 5-fold cross-validation was also used to assess the viability of the proposed model, a summary of which is available in Table 5. The model reached the accuracy above 86% in all the folds, which proves its stability.

**Table 4:** Final performance metrics of the hybrid model.

Metrics	Value
Accuracy	0.8355
Precision	0.84333
Recall	0.8241
F1-Score	0.83361
AUC	0.90938
Sensitivity	0.98241

**Table 5:** Cross-validation metrics for the proposed hybrid model.

Fold	Accuracy	Precision	F1-score
Fold 1	0.8800	0.8543	0.8841
Fold 2	0.8636	0.8421	0.8678
Fold 3	0.8893	0.8744	0.8914
Fold 4	0.8730	0.8620	0.8749
Fold 5	0.8823	0.8612	0.8856

### Performance analysis of proposed hybrid model with ACO

**Fig. 11:**
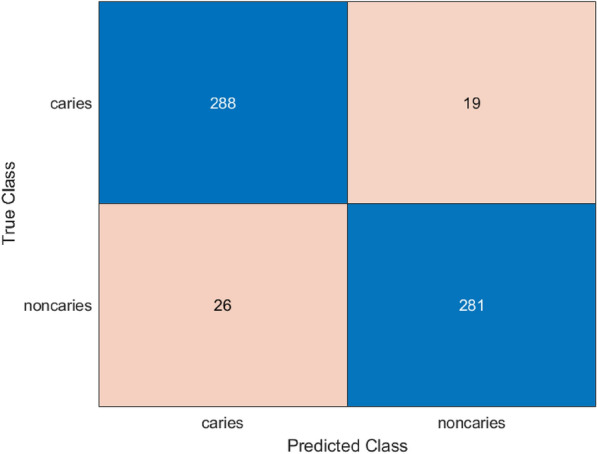
Confusion matrix for proposed hybrid model with ACO.

**Fig. 12:**
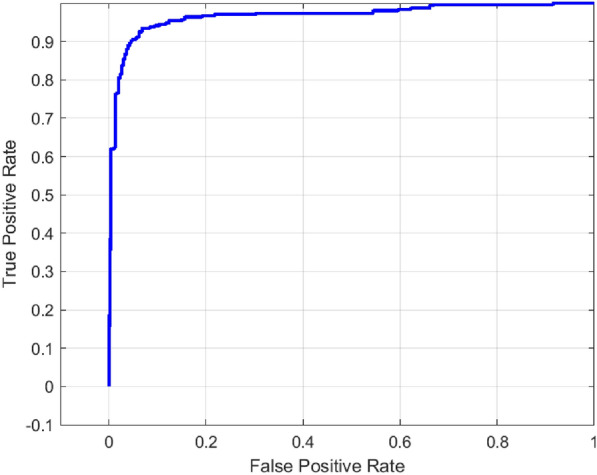
ROC curve for proposed hybrid model with ACO.

In applying the ACO to the hybrid model, there was excellent performance compared to the other models because of its characteristic of searching for the ideal hyperparameters to further assist in the effective and accurate classification. Hybrid model that has been trained on a binary-classified prediction of dental caries on enhanced X-ray images was tuned through hyperparameter adjustment with the help of the ACO technique. The configuration which performed the best consisted of learning rate momentum, mini-batch size as shown in Table 6. Figures 11 and 12 represent the confusion matrix and ROC curve for the proposed hybrid model with ACO .

The overall performance of the suggested system satisfies the good results obtained on the last evaluation on test collection, as it can be demonstrated in Table 7. The model exhibits significant improvements with an accuracy of 92.67%, a precision of 93.67% with a recall of 91.53% and AUC of 96.79%.

**Table 6:** Best hyperparameters obtained via ant colony optimization.

Hyperparameter	Value
Learning rate	0.00455
Momentum	0.830
MiniBatch size	50

**Table 7:** Final performance metrics of the hybrid model with ACO.

Metrics	Value
Accuracy	0.92671
Precision	0.93667
Recall	0.91531
F1-Score	0.92586
AUC	0.9679
Sensitivity	0.91531

Cervical burnout is a radiographic artifact that would present as a radiolucent band at the cervical area, frequently resembling dental caries and resulting in false positives in the case of automated classification^38^. Explainable AI approaches, such as Grad-CAM (Gradient-weighted Class Activation Mapping), provide visual heatmaps that show which elements of an image a model depends on the most to produce a forecast. Grad-Cam computes the gradient of the desired result (e.g., the presence of caries) flowing into the final convolutional layer of a neural network and applies it to weight feature map importance^39^. The process generates localization maps that indicate where the model gazes during decision making in order to confirm that the focus is placed in an anatomically relevant region as opposed to such artifacts as cervical burnout.

**Fig. 13:**
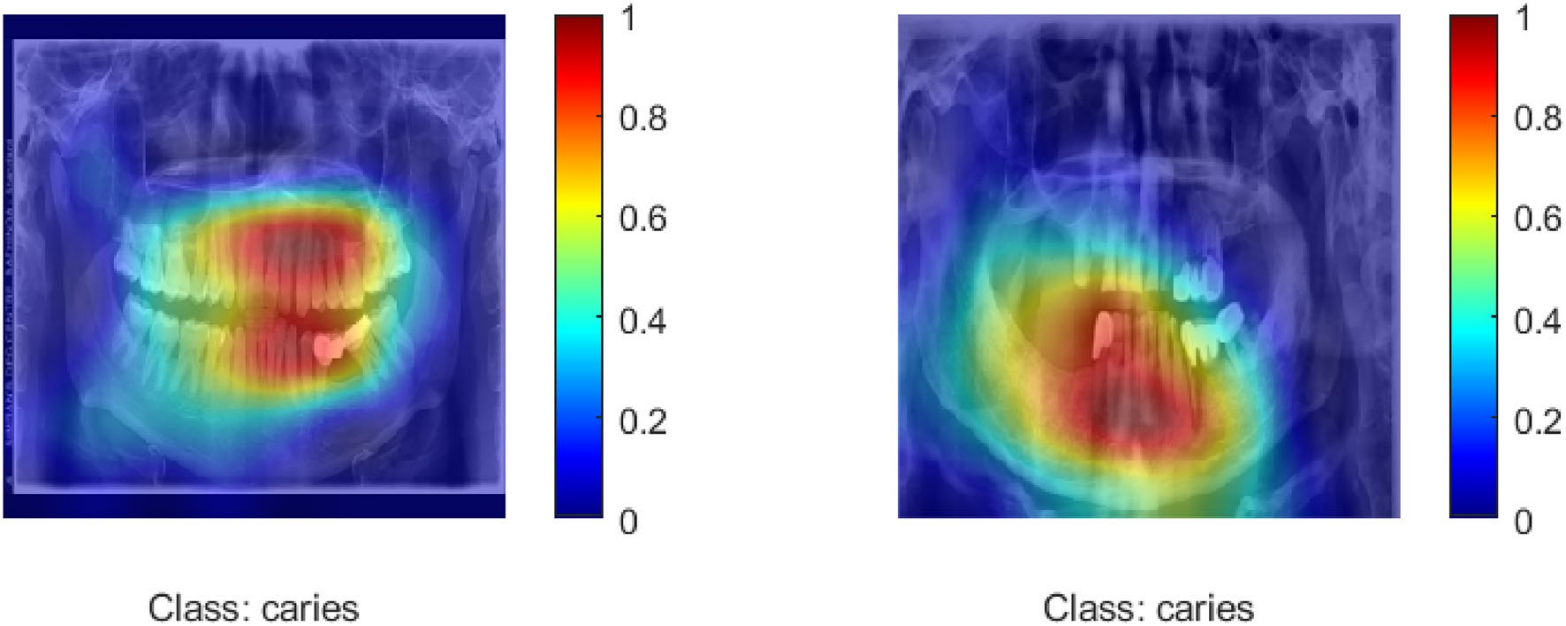
Grad-Cam heatmap images highlighting caries effected regions in panoramic radiographic images.

The Fig.13 demonstrates Grad-CAM heatmaps overlaid on dental radiographs, which gives a visual representation of where the hybrid network concentrates to identify dental caries. The heat maps apply a color gradient with red and orange coloration indicating areas with most model activity meaning that they are more relevant and have more likely carious lesions present. Colors like blue and green indicate areas with least influence on the classification decision of the model. This type of visualization makes the interpretations more understandable, as the spatial areas contributing to the greatest output of the network can be seen, and clinicians can ensure that the AI model focuses on the anatomically and diagnostically relevant areas, thereby fostering trust and transparency in AI-assisted dental diagnosis.

### Comparative study

Table 8 provides an overview of a comparative analysis of our proposed model with respect to the previous works that focused on classification and detection of dental caries with the use of various kinds of radiographs. It can be observed that our proposed model by the integration of a hybrid model and the ACO, has competitive performance in caries detection as compared to other existing strategies.

**Table 8:** Comparative study of caries detection approaches with performance metrics.

Ref	Year	Identification type	Dataset information	Models used	Performance metrics
^40^	2021	Caries	434 Photographic images	SqueezeNet	Accuracy-88.0%, F1-score-68.0%
^41^	2021	Caries	Bitewing radiographs, 304 images	U-Net	Recall-65.02, F1-score-64.14%
^42^	2022	Caries	Intraoral images, 2417 images	MobileNetV2	Accuracy-92.5%, specificity-94.3%
^43^	2022	Caries	Oral photographs, 3932 images	ConvNet	AUC-85.65%, sensitivity-81.90%
^44^	2023	Caries	Panoramic X-rays, 116 images	NasNetMobile	Recall-81.0%
^45^	2024	Non-normal teeth	Panoramic X-rays, 1008 images	Mask R-CNN + Attention	Accuracy-85.2%
^46^	2024	Caries	Periapical radiographs, 4278 images	ResNet + SAM	Accuracy-88.5%, AUC-95.4%
^47^	2024	Caries	Bitewing radiographs, 425 images	Mask-RCNN, swin transformer	Sensitivity-74.2%, precision-67.6%, F1-score-68.9%
^48^	2025	Caries	Intraoral scans	Attention U-Net	Sensitivity-71%, precision-66%
Proposed method	2025	Caries	Panoramic radiographs	Hybrid model (MobileNetV2+Shuffle-Net) + ACO	Accuracy-92.67%, precision-93.66%, recall-91.53%, F1-score-92.58%, AUC-96.79%, sensitivity-91.53%

## Conclusion

This paper presents an elaborate method for categorizing dental caries using panoramic radiographic images. First clustering-based method was used to resolve the issue of class imbalance in the dataset, and then edge detection was used with Sobel-Feldman to increase the significance of specific features through preprocessing. Although MobileNetV2 and ShuffleNet as individual deep learning models were used, they did not perform well when used alone. To make the best use of the characteristics of both architectures, a hybrid model was built, uniting the advantages of the architectures. This was done by further optimization using the ACO algorithm within the hybrid framework that produced quite high performance values. The ACO-strengthened hybrid model showed better classification accuracy, due to its strong global search strategy as well as optimal parameter settings, hence confirming its promise in automated dental diagnosis systems.The accuracy scores of the individual models were moderate, where MobileNetV2 and ShuffleNet scored 76% and 82% respectively. Conversely, the proposed hybrid model has shown a better accuracy rate of 83%, and by combining the model with ACO, the level of accuracy was further improved to 92% indicating the efficiency of the hybrid model. The present work is restricted to classifying and detecting caries in X-ray images without assessing severity levels or specific tooth positions. Future works should explore hybrid approaches that analyze deeper dentin and pulp regions for improved diagnostic precision. Expanding to multi-class classification, segmentation, and validation on external datasets using different image modalities can strengthen the model’s reliability and clinical relevance.

## Data Availability

The data is taken from a publicly available dataset, which can be accessed through https://www.kaggle.com/ datasets/lokisilvres/dental-disease-panoramic-detection-dataset
